# Supplementary material for: The interactome of intact mitochondria by cross-linking mass spectrometry provides evidence for coexisting respiratory supercomplexes
Source: Mol Cell Proteomics. 2017 Dec 8;17(2):216–32. doi: 10.1074/mcp.RA117.000470 (PMC5795388; doi:10.1074/mcp.RA117.000470)
Supplement: Supplemental Data [file supp_RA117.000470_133922_0_supp_23978_fzffwf.zip › spectra_annotation/mito_DR_spectra_annotation/31-1-8-1-8-1.pdf]

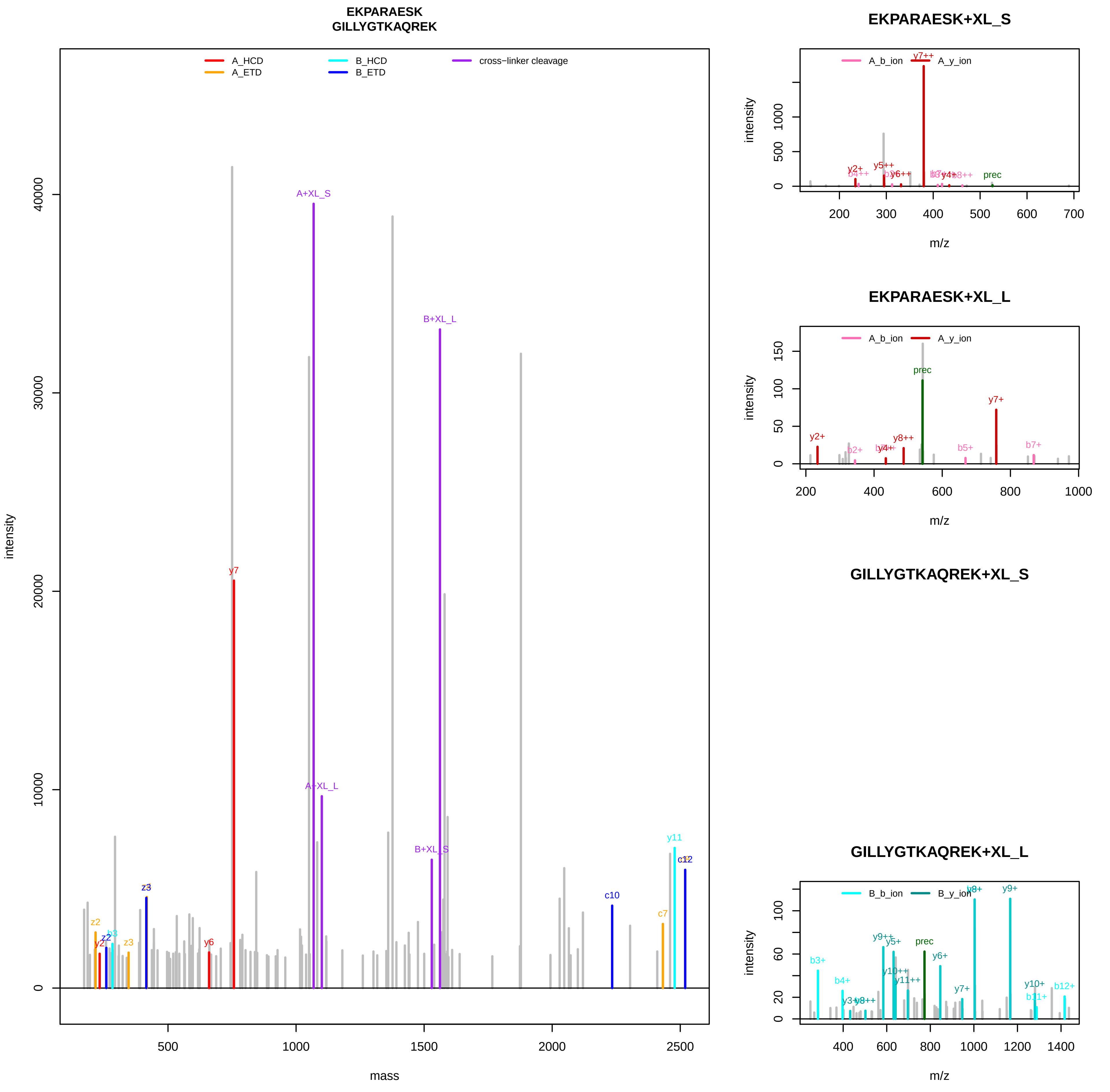
EKPARAESK
GILLYGTKAQREK
A_HCD
A_ETD
B_HCD
B_ETD
cross−linker cleavage
A+XL_S
B+XL_L
A+XL_L
B+XL_S
y7
y6
y2
z2
z3
b3
c10
c7
y11
c12
intensity
mass
EKPARAESK+XL_S
A_b_ion
A_y_ion
y7++
y2+
y5++
y6++
prec
m/z
EKPARAESK+XL_L
y7+
y8++
y2+
b2+
b5+
b7+
GILLYGTKAQREK+XL_S
GILLYGTKAQREK+XL_L
B_b_ion
B_y_ion
y9+
y9++
y5+
y6+
y7+
b3+
b4+
y10++
y11++
y10+
b11+
b12+
